# Reply to the letter by Calle and Mpotos: Why not try harder to prove that automated external defibrillators save lives?

**DOI:** 10.1007/s12471-019-1249-y

**Published:** 2019-02-28

**Authors:** J. Nas, J. Thannhauser, M.-J. de Boer, J. L. Bonnes, M. A. Brouwer

**Affiliations:** Department of Cardiology, Radboud University Medical Centre, Nijmegen, The Netherlands

We would like to thank Calle and Mpotos for their interest in the topic of automated external defibrillators (AEDs) and their valuable comments [1] on our article ‘Changes in automated external defibrillator use and survival after out-of-hospital cardiac arrest (OHCA) in the Nijmegen area’, published in the December issue of *Netherlands Heart Journal*.

Like many other OHCA registries our study has several limitations, i. e. patient selection and the absence of AED time intervals, both of which we address in the ‘Limitations’ section of our article. Furthermore, we acknowledge the comment made by Calle and Mpotos that it is important to mention how many of the patients actually received an AED shock. In fact, this is why we reported not only the increase in AED attachment, but also specifically focused on the subgroup of patients receiving an AED shock. In addition, we demonstrated an independent association between defibrillation with an AED and outcome in our study cohort.

Calle and Mpotos mention a randomised controlled trial (RCT) to avoid methodological limitations. Indeed, an RCT remains the gold standard to provide robust evidence of benefit of any intervention and thus far RCTs on AED use are scarce [2]. However, it has been demonstrated that survival following OHCA decreases by ~10% with every passing minute without intervention, and early defibrillation is therefore a major predictor of a favourable outcome [3]. This, and the high survival rates in settings where AEDs were readily available, prompts the question whether randomisation between AED and no AED would be ethical [4]. An RCT on dispatcher-activated lay-person interventions is underway, and those results may provide additional insight into the effectiveness of public access defibrillation (NCT02010151; https://clinicaltrials.gov/).

As noted previously, most of the evidence on public access defibrillation stems from observational research, which limits inferences regarding causality [2, 4]. However, as Calle and Mpotos also acknowledge, observational research may very well be of value to evaluate resuscitative interventions in general, and the use of AEDs in particular. As high-quality observational research requires high-quality data, we underscore the need for uniform, detailed data collection in the setting of multicentre or national registries. In this context, we support the efforts of the Dutch Heart Foundation to unite experts in the field and gather data from multiple regions. Awaiting further developments, several research groups have presented the available data from the Netherlands, and we eagerly await the initiatives of Calle and Mpotos to present theirs.
